# Transcriptome analysis of renal ischemia/reperfusion (I/R) injury in BAFF and BAFF-R deficient mice

**DOI:** 10.1371/journal.pone.0291619

**Published:** 2023-09-26

**Authors:** Tamara Möckel, Sebastian Boegel, Andreas Schwarting

**Affiliations:** 1 Department of Internal Medicine I, Division of Rheumatology and Clinical Immunology, University Medical Center of the Johannes Gutenberg University, Mainz, Germany; 2 Center for Rheumatic Disease Rhineland-Palatinate GmbH, Bad Kreuznach, Germany; Zagazig University, EGYPT

## Abstract

Acute kidney injury (AKI) accompanies with high morbidity and mortality. Incomplete renal recovery can lead to chronic and finally end-stage kidney disease, which results in the requirement of lifelong dialysis or kidney transplantation. Consequently, finding predictive biomarker and therefore developing preventive therapeutic approaches is an urgent need. For this purpose, a better understanding of the mechanism underlying AKI is necessary. The cytokine BAFF (B cell activating factor) is related to AKI by supporting B cells, which in turn play an important role in inflammatory processes and the production of antibodies. In our study, we investigated the role of BAFF and its receptor BAFF-R in the early phase of AKI. Therefore, we performed the well-established ischemia/reperfusion (I/R) model in BAFF (B6.129S2-*Tnfsf13b*^*tm1Msc*^/J) and BAFF-R (B6(Cg)-*Tnfrsf13c*^*tm1Mass*^/J) deficient mice. Transcriptome of ischemic and contralateral control kidneys was analyzed and compared to wildtype littermates. We detected the upregulation of Lcn2, Lyz2, Cd44, Fn1 and Il1rn in ischemic kidneys as well as the downregulation of Kl. Furthermore, we revealed different expression patterns in BAFF and BAFF-R knockout mice. Compared to wildtype littermates, up- and downregulation of each investigated gene were higher in BAFF-R knockout and lower in BAFF knockout. Our findings indicate a positive impact of BAFF knockout in early phase of AKI, while BAFF-R knockout seems to worsen I/R injury. In addition, our study shows for the first time a remarkable renal upregulation of Lyz2 in a murine I/R model. Therefore, we consider Lyz2 as conceivable predictive or early biomarker in case of I/R and AKI.

## Introduction

B cell activating factor (BAFF, TNFSF13B) is a cytokine of the tumor necrosis factor (TNF) family, which was first described by Schneider *et al*. in 1999 [1]. It plays an important role for activation, differentiation and survival of B cells [1, 2]. In addition, BAFF is closely linked with autoimmunity and exerts both inflammatory and regulatory effects [reviewed in 3]. Contrary to the long-standing assumption that only myeloid cells are expressing BAFF, a wide variety of cells is now known to be a source of this cytokine [reviewed in 4]. BAFF is a type II membrane-bound protein [5], which is cleaved by furin and released in its biological active form as a homotrimer [6]. Signaling is possible via three different receptors: BAFF receptor (BAFF-R, TNFRSF13C), mediating survival signals [7–9], BCMA (B cell maturation antigen, TNFRSF17), involved in differentiation and survival of long-lived bone marrow plasma cells and plasmablasts [10, 11] and TACI (transmembrane activator and cyclophilin ligand interactor, TNFRSF13B), required for negative regulation, class-switch recombination and T cell-independent antibody response [12–15]. Interestingly, the binding of one BAFF trimer to BAFF-R leads to no or only very low signal. Interaction with another BAFF trimer, bound to BAFF-R, via flap region is required for signal transduction, which shows that binding to and activation of BAFF-R are separate processes [16]. Upregulation of BAFF has been reported in acute kidney failure, transplantation and graft rejection [17–22].

Acute kidney injury (AKI), used synonymously with acute renal failure (ARF), ranges from minor to complete loss of kidney function and therefore accompanies with high morbidity and mortality. In case of incomplete recovery, patients can develop chronic and finally end-stage kidney disease, resulting in the requirement of a lifelong dialysis or kidney transplantation. Ischemia/reperfusion (I/R) is an appropriate model for AKI. The reasons for damage evoked by I/R are oxidative stress and the production and reduced scavenging capacity of reactive oxygen species (ROS) at the same time [23]. It is erroneous to assume that only ischemia is responsible for the injury. Ischemia first initiates the injury, followed by cell death, but reperfusion causes additional damage [24–26]. Thus, I/R contributes to renal dysfunction, which is associated with acute renal failure [27, 28] and necrotic pathways [29]. Venkatachalam *et al*. [30] showed that the proximal section of tubular epithelial cells (TECs) is vulnerable to I/R injury. The early loss of TECs after I/R is caused by the activation of death receptor dependent and mitochondrial pathways [31]. I/R injury is the major cause of acute renal failure in native [27, 32] as well as transplanted kidneys [33]. In transplanted kidneys I/R injury leads to impaired graft function [34] and thereby to a higher risk of graft rejection [35, 36]. Immune cells play a leading role in AKI, which can also be caused by sepsis or toxins instead of ischemia events. B cells seem to have an ambivalent role. Burne-Taney *et al*. [37] postulated a pathogenic function of B cells in I/R injury. B cell-deficient mice, unable to develop peripheral mature B cells, were protected from I/R injury [37]. The transfer of serum from wildtype mice restored the I/R injury phenotype [37]. Furthermore, due to natural Ab IgM, which bind to mesangium, B cells act pathogenic after I/R [38]. At the same time B cells produce anti-inflammatory Interleukin (IL)-10, which has a protective effect [38]. Thus, the role of BAFF in I/R is not completely understood.

It is shown, that renal tubular atrophy and thereby tubular proliferation is altered by kidney infiltrating B cells after I/R [39]. The threshold for induction of fibrosis and progression of atrophy amounts an ischemia time of 35 minutes [40]. I/R injury exhibits two phases. The first phase is seen within the first three days, marked by immediate response to initial injurious trigger, which can be seen in a peak of inflammation, injury and cell death markers. In the second phase, which begins on day three of reperfusion, inflammation, injury and cell death markers decrease, but fibrosis markers peak [40].

In case of graft rejection retransplantation is required [17] and for end-stage renal disease patients transplantation is the best treatment option [41]. The main problem of transplantations is graft rejection, also under immunosuppression. Applied drugs are focused on T cells and thereby preventing acute rejection. These drugs are inefficient preventing chronic rejections [42, 43]. Serum levels of donor specific antibodies (DSA) and C4d staining of kidney biopsies are still the gold standards, even though both methods are not ideal [44]. Applied immunotherapies to suppress the immune system and prevent rejection increase susceptibility for infection and cancer, have severe side effects and are often nephrotoxic. Altogether leads to dysfunction of the allograft. Another difficulty is the fact, that antibody-mediated rejection (AMR) often do not show the typical phenotypes and clinical manifestations, which leads in a delay of diagnosis and treatment [17]. Actual the 10 years graft survival rate amounts 56% [45], AMR causes 65% of late failures [46]. Key players with regard to rejection are B cells, which are essential contributors to transplant tolerance, produce antibodies against donor human leukocyte antigen (HLA) and non-HLA antigens, activate T cells, produce cytokines, promote inflammation and are involved in the development of tertiary lymphoid organs [reviewed in 47]. In turn, B cells are supported by BAFF, which plays a critical role in the process of antibody-mediated immune-response. Consequently, BAFF is considered as biomarker to predict graft rejection. Levels of BAFF are higher in kidneys with abnormal function and correlate with anti-HLA I and II antibodies [48]. High BAFF protein level and the development of *de novo* HLA antibodies [20] are associated with acute AMR [18]. BAFF was detected in renal transplant biopsies with acute rejection [21]. In case of antibody-incompatible transplantation elevated BAFF serum levels, before removal of antibodies and transplantation, are associated with increased risk of AMR [18]. Although in antibody-compatible transplantation high BAFF level represent a great risk for developing DSA and high BAFF-R mRNA levels predict graft dysfunction [20]. In renal allografts with chronic rejection high BAFF mRNA as well as protein level and BAFF-R positive B and T cells were detected [19, 22]. Serum as well as mRNA BAFF level are increased in chronic antibody-mediated rejection and stable patients compared to healthy controls [17]. But there is no statistically significant difference between stable and chronic antibody-mediated rejection patients, which leads to the assumption, that BAFF is not a useful biomarker for predicting graft rejection [17]. TACI, one receptor of BAFF and overexpressed in patients with chronic antibody-mediated rejection, is maybe a potential biomarker for distinguishing chronic antibody-mediated rejection from stable patients [17]. In addition, Belimumab, a humanized anti-BAFF antibody is successfully used in the treatment of the classic autoimmune disease Systemic Lupus Erythematosus (SLE) and associated Lupus Nephritis [49–51]. However, no data are available on possible effects of Belimumab therapy in ischemic renal disease, neither in patients nor experimental models.

**Fig 1:**
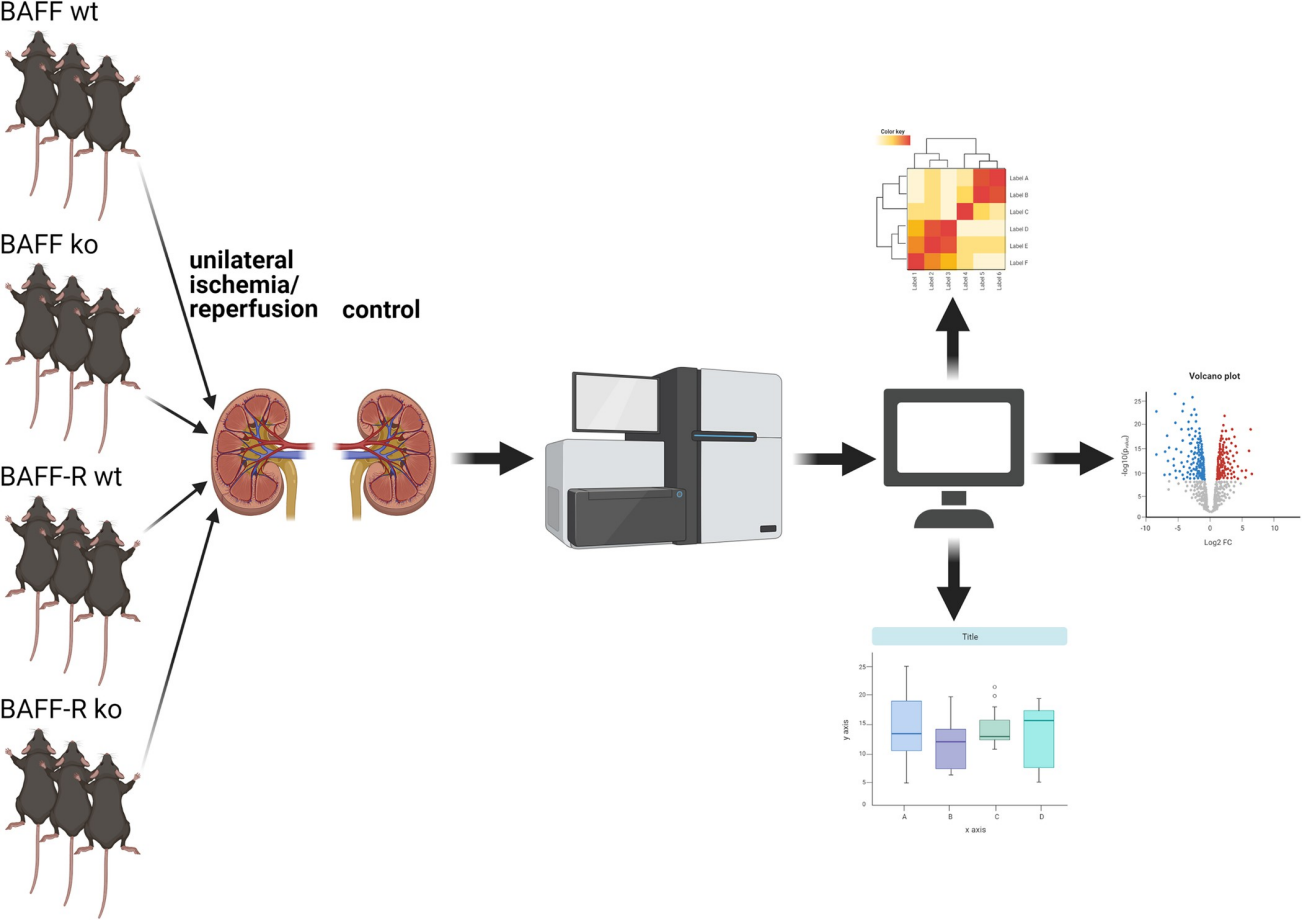
Experimental study design. Unilateral ischemia/reperfusion (I/R) of the left kidney was performed in BAFF KO (B6.129S2-*Tnfsf13b*^*tm1Msc*^/J) and BAFF-R KO (B6(Cg)-*Tnfrsf13c*^*tm1Mass*^/J) mice, followed by transcriptome analysis of ischemic and contralateral control kidneys compared to appropriate wildtype littermates. Created with BioRender.com.

To unravel the effects of BAFF and its interaction with BAFF-R in the development of acute kidney injury, we used the well-established ischemia/reperfusion model in BAFF and BAFF-R knockout mice. We are not aware of any study investigated the BAFF/BAFF-R system in a murine kidney I/R model so far. Instead of studying genes already known to be upregulated in the I/R model, we performed a comprehensive analysis of all up- and downregulated genes by high throughput RNA sequencing technology. Fig 1 shows the transcriptome approach analyzing the ischemic as well as contralateral kidneys. In particular the impact of the BAFF and BAFF-R knockout will be evaluated with regard to altered extent of renal damage and potential protective effects.

## Materials and methods

### Ethical statement

Housing, breeding, handling and experimental procedures were conducted in accordance with EU Directive 2010/63/EU as well as the German Animal Welfare Act and were approved by the local authority (Landesuntersuchungsamt Rhineland-Palatinate, reference number 23 177-07/ G 18-1-024).

### Laboratory animals

B6.129S2-*Tnfsf13b*^*tm1Msc*^/J, common name BAFF KO (RRID: IMSR_JAX:010572) [52, 53] and B6(Cg)-*Tnfrsf13c*^*tm1Mass*^/J, common name BAFF-R KO (RRID: IMSR_JAX:007212) [54], were purchased form The Jackson Laboratory (Maine, USA). Mice were housed in individually ventilated cages (IVCs) in a specific-pathogen-free (SPF) unit under standardized conditions (12/12 hours light/dark cycle, room temperature 22 ±2°C, humidity 50–70%). Autoclaved food and water were supplied *ad libitum*. For experimental procedure, eight weeks old female BAFF KO and BAFF-R KO mice as well as their wildtype littermates were housed in filtertop cages in a conventional unit under standardized conditions (12/12 hours light/dark cycle, room temperature 20 ±2°C, humidity 50–70%).

### Unilateral ischemia/reperfusion (I/R)

Borgal 24% (License number 6489082.00.00, Virbac, Carros, France), a mixture of the antibiotics sulfadoxine and trimethoprime, was administered preventive via animals‘ drinking water (final 0.048%) as of three days before intervention until the end of experiment. For analgesia Buprenorphine (Temgesic, PZN 345928, Indivior, Virginia, USA), 0.1 mg/kg body weight, was injected i.p. 30 minutes before surgery. Isoflurane (PZN 09714675, Piramal Critical Care, Pennsylvania, USA) was used for anesthesia, induction in chamber with 2.75% by volume and during surgery via inhalation mask with 1.8% by volume. Mice were placed in abdominal position on a heating pad to maintain body temperature during intervention and limbs were fixed, except one. Eyes were protected from drying out by application of eye ointment. After reaching tolerance, stage III of surgical anesthesia according to Guedel [55], checked by unresponsiveness to pinch at toe interdigit of not fixed limb, surgical site was shaved and disinfected with 70% ethanol. With a first cut via left flank incision as small as possible, skin and then peritoneum were opened. Left kidney was exteriorized by using a curved glass capillary. Afferent artery was disconnected by using micro aneurysm clip (Cat. No. 61–0186, Harvard Apparatus, Massachusetts, USA) for 45 minutes. During ischemia time left kidney was relocated into retroperitoneal space and surgical site kept moist with a 0.9% physiological sodium chloride solution containing benzyl alcohol as bacteriostatic agent. For removement of atraumatic hemostat, left kidney was again exteriorized and then relocated into retroperitoneal space. Peritoneum was closed with single-button suture, skin with wound clips (Cat. No. 427631, Clay Adams, Connecticut, USA). Analgesic was applied once again during last control at surgery day, on the following days only if necessary. Reperfusion time amounted three days. After this time, mice were sacrificed by cervical dislocation and ischemic as well as contralateral kidney removed, partly shock frozen and stored at -80°C until RNA isolation.

### RNA isolation

Shock frozen kidney tissue was homogenized in Lysis Solution RL (kit further on) by using Tissue Lyser LT (Qiagen N.V., Venlo, Netherlands). Isolation of total RNA from homogenized and lysed kidney tissue was performed with innuPREP RNA Mini Kit (Cat. No. 845-KS-2040250, Analytik Jena GmbH, Jena, Germany) according to manufacturer‘s manual. RNA concentration and purity was calculated with NanoDrop 2000 (Thermo Fisher Scientific Inc., Massachusetts, USA).

### RNA sequencing

Steps described in this section were completely performed by StarSEQ GmbH (Mainz, Germany). Measurement of RNA integrity number (RIN) was performed with Agilent RNA 6000 Nano Kit (Cat. No. 5067–1511) and Agilent 2100 Bioanalyzer (kit and device from Agilent, California, USA). RNA concentration was defined by using Qubit RNA High Sensitivity (HS) Assay Kit (Cat. No. Q32855) and Qubit 4 Fluorometer (kit and device from Thermo Fisher Scientific Inc., Massachusetts, USA). Library preparation was done with NEBNext Ultra II Directional RNA Library Prep Kit for Illumina with unique dual index primer pairs (Cat. No. E7760L, New England BioLabs Inc., Massachusetts, USA). Concentration and quality of library preparation was measured with Qubit 4 Fluorometer by using Qubit dsDNA HS Assay Kit (Cat. No. Q32854, Thermo Fisher Scientific Inc., Massachusetts, USA) and QIAxcel Instrument with QIAxcel ScreenGel 1.5.0 Cartridge (Qiagen N.V., Venlo, Netherlands). RNA sequencing (paired-end, 150 bp read length, approximately 25 million reads per sample (usually more)) was performed with Illumina NextSeq 2000 (Illumina, California, USA). Data was provided as FASTQ files.

### Sampling and exclusion criteria

In the following bioinformatical analyses, six samples of each strain were investigated (BAFF and BAFF-R). Per strain three ischemic or rather contralateral knockout kidneys were compared to three ischemic or rather contralateral wildtype kidneys.

Samples clustered into two groups, ischemic and contralateral kidneys. If ischemia/reperfusion was not sufficient, recognized by an expression pattern of the ischemic kidney similar to the contralateral kidney, respective sample was excluded from further analyses.

### Bioinformatical and statistical analyses

Paired-end RNA Seq data were analyzed on a MacBook with kallisto 0.46.1 [56] using Ensemble Transcriptome v96 *mus musculus* (https://github.com/pachterlab/kallisto-transcriptome-indices/releases) as reference, manually deleting transcript sequences from hemoglobin chains. Analysis is carried with R (version 4.1.2) using DeSeq2 [57] for differential gene expression analysis and principal component analysis (PCA). DeSeq2 uses the read counts for each gene in each sample, builds a generalized linear model of the negative binomial family and after several mathematical steps including normalization, estimation of gene-wise dispersion, shrinkage estimation of logarithmic fold changes, Fisher estimation, Wald test and Cook’s distance for outlier detection and adjusting for multiple testing using the procedure of Benjamini and Hochberg [58], the tool outputs a log2foldchange and an adjusted p-value for each gene, reflecting the variability of the expression of this gene within a group and between the groups (i.e. differentially expressed between the groups). All mathematical details are described in [58]. Volcano Plots are made with Bioconductor package "Enhanced Volcano" [59]. Figures are made with ggplot2 [60]. All scripts, gene expression data and supplement tables to reproduce the figures and analysis are available at https://github.com/sebboegel/moeckel2022.

## Results

**Fig 2:**
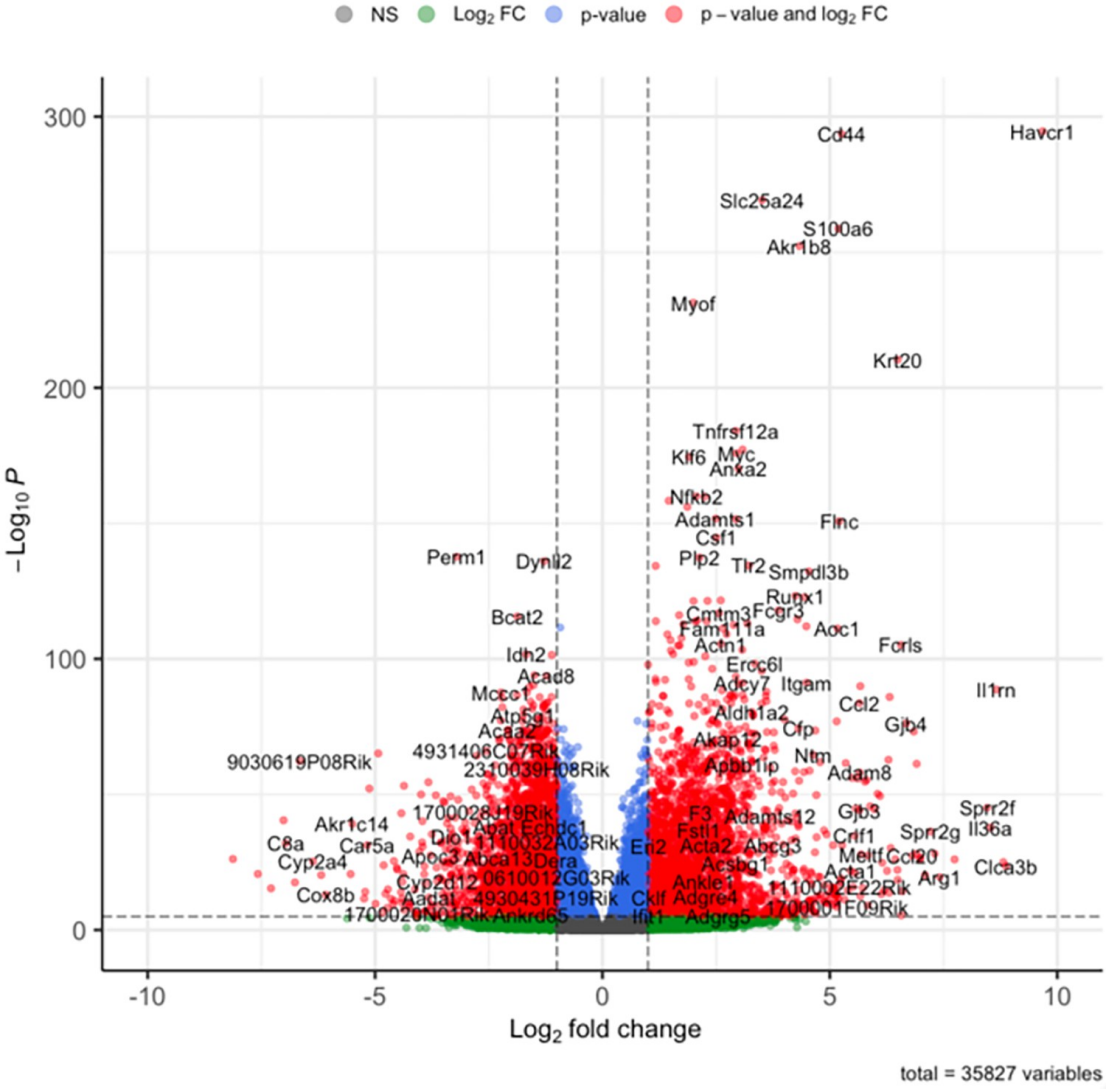
Volcano plot visualizing differentially regulated genes in ischemic versus contralateral kidneys without distinction between BAFF (B6.129S2-*Tnfsf13b*^*tm1Msc*^/J) and BAFF-R (B6(Cg)-*Tnfrsf13c*^*tm1Mass*^/J) strain.

**Fig 3:**
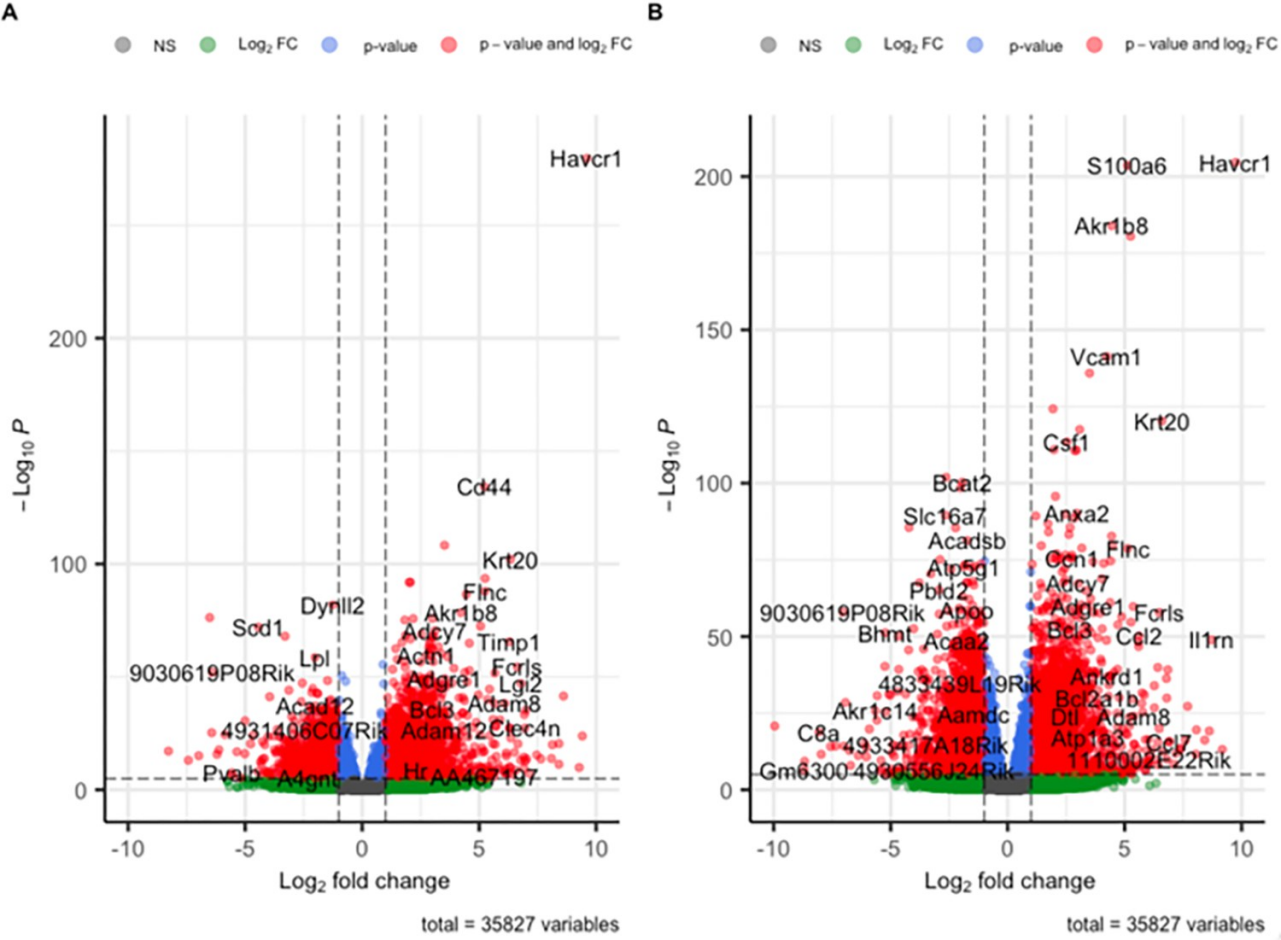
Volcano plot visualizing differentially regulated genes in ischemic versus contralateral kidneys. A) BAFF (B6.129S2-*Tnfsf13b*^*tm1Msc*^/J) strain. B) BAFF-R (B6(Cg)-*Tnfrsf13c*^*tm1Mass*^/J) strain.

**Fig 4:**
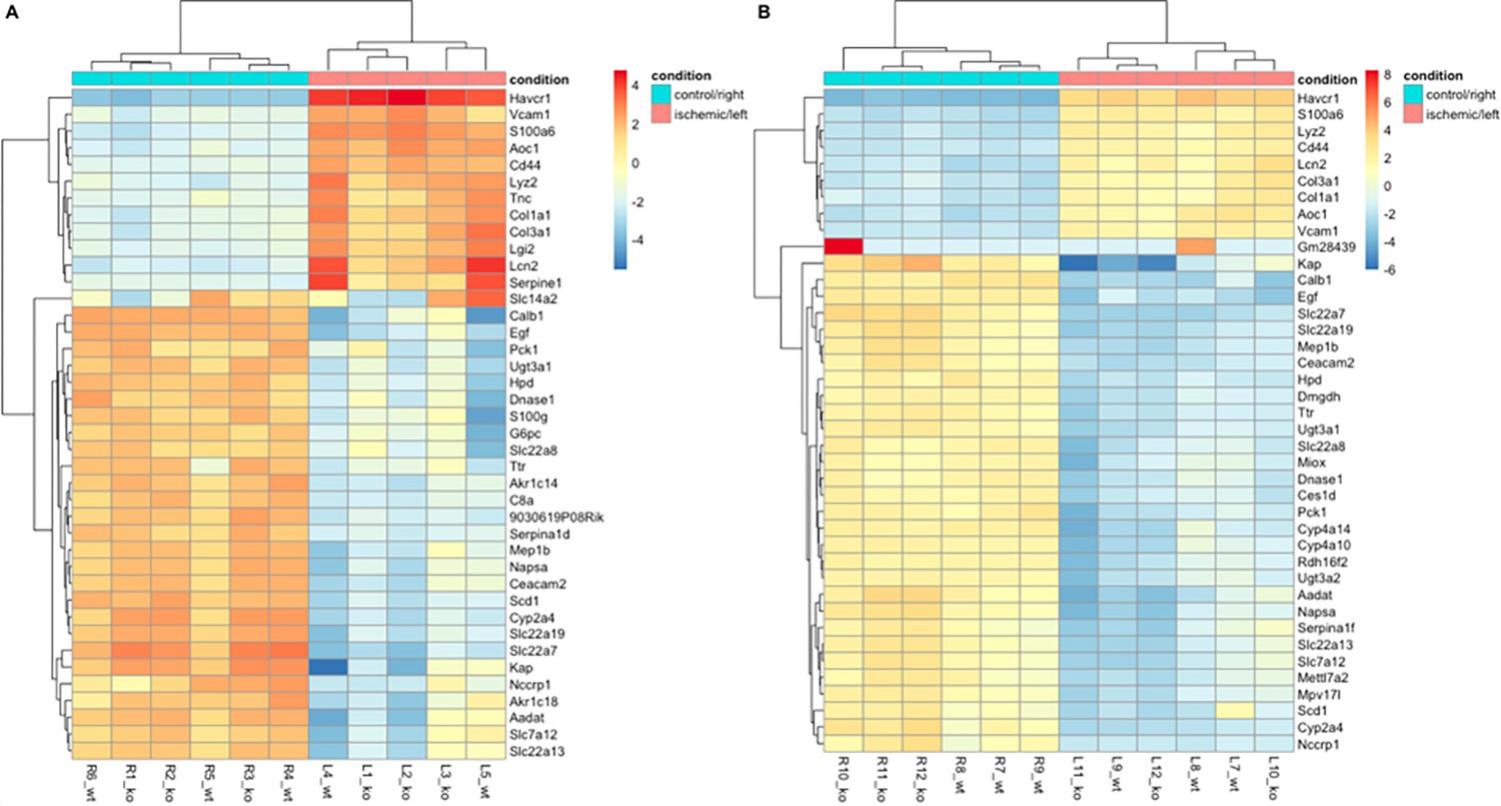
Heatmap visualizing top 40 differentially expressed genes in ischemic versus contralateral kidneys. A) BAFF (B6.129S2-*Tnfsf13b*^*tm1Msc*^/J) strain. B) BAFF-R (B6(Cg)-*Tnfrsf13c*^*tm1Mass*^/J) strain. R: right kidney (contralateral control), L: left kidney (unilateral I/R), ko: knockout, wt: wildtype.

Three days after unilateral ischemia/reperfusion injury a total of 1905 genes were significantly differentially regulated in ischemic kidneys compared to contralateral kidneys, thereby 1364 up- and 541 downregulated (defined as a log2foldchange > 1, < -1 resp, adjusted p-value < 0.05, see source code, S1 File and Fig 2). We found more up- than downregulated genes in ischemic kidneys and vice versa in contralateral kidneys regardless of the underlying mouse strain (Fig 3). Of these genes, we find Havcr1 (p < 1.49922764165841e-76), Lcn2 (p < 3.44791875648706e-10), Lyz2 (p < 9.94678438201453e-33) and Cd44 (p < 4.32218440489859e-34) amongst the top 40 differentially expressed genes for BAFF (Fig 4A) and BAFF-R (Fig 4B) strains. The four genes were upregulated in both strains and thereby in knockout as well as wildtype mice (Fig 4).

Expression pattern of the left kidney of sample L6_wt was similar to contralateral kidneys and therefore ischemia/reperfusion injury was not sufficient (S1 Fig). Consequently, sample L6_wt was not included in further analyses. However, the remaining samples clustered into two groups, ischemic and contralateral kidneys.

**Fig 5:**
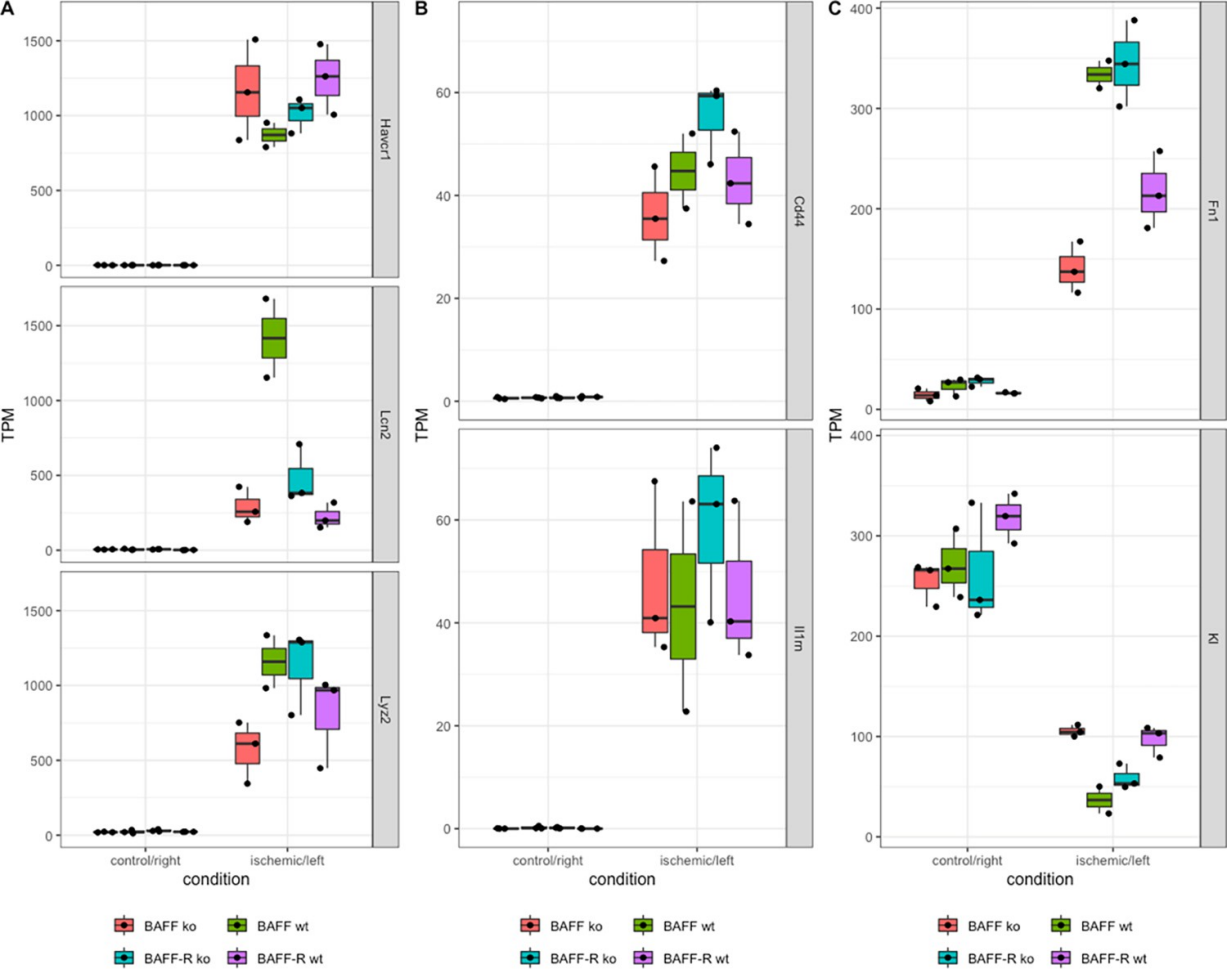
Transcripts per million (TPM) of analyzed genes of ischemic kidneys compared to contralateral control in BAFF (B6.129S2-*Tnfsf13b*^*tm1Msc*^/J) and BAFF-R (B6(Cg)-*Tnfrsf13c*^*tm1Mass*^/J) strain, in each case distinguished between knockout (ko) and wildtype (wt) mice. A) Havcr1: Hepatitis A virus cellular receptor 1, Lcn2: Lipocalin-2, Lyz2: Lysozyme 2. B) Cd44: CD44 antigen, Il1rn: Interleukin-1 receptor antagonist. C) Fn1: Fibronectin 1, Kl: Klotho. Each point shows one sample.

Havcr1 was consistently upregulated in ischemic kidneys of knockout and wildtype mice in both strains (Fig 5A). However, BAFF knockout kidneys showed higher Havcr1 expression compared to wildtype littermates, whereas in the BAFF-R strain the expression of Havcr1 was higher in wildtype than knockout kidneys. Comparing both strains, Havcr1 expression was higher in wildtypes of BAFF-R strain than of the BAFF strain. For all contralateral kidneys no expression of Havcr1 was detected.

Expression level of Lcn2 was lower in BAFF knockout kidneys compared to wildtype littermates (Fig 5A). BAFF-R knockout kidneys showed a slightly higher Lcn2 expression than wildtype kidneys. Lcn2 expression level in ischemic wildtype kidneys of BAFF strain is higher compared to BAFF-R strain wildtypes. No expression was detected in the contralateral kidneys of both strains, regardless of genotype.

Lyz2 expression was lower in BAFF knockout kidneys compared to wildtype littermates (Fig 5A). Kidneys of BAFF-R knockout mice showed higher Lyz2 expression level compared to their wildtype littermates. In contralateral kidneys, knockout as well as wildtype of both strains, there was no Lyz2 expression detectable.

The expression of Cd44 was upregulated in ischemic kidneys compared to contralateral controls in both strains independent of the genotype (Fig 5B). Cd44 expression was lower in BAFF knockout kidneys and higher in BAFF-R knockout kidneys compared to the respective wildtypes. Ischemic wildtype kidneys of both strains showed similar Cd44 expression levels.

Another upregulated gene due to ischemia/reperfusion injury, Fn1, showed a lower expression level in BAFF knockout kidneys compared to wildtype kidneys, whereas Fn1 expression was higher in BAFF-R knockout than wildtype kidneys (Fig 5C). No Fn1 expression was detected in contralateral kidneys of both strains. Ischemic wildtype kidneys of BAFF strain showed higher expression levels for Fn1 than wildtype kidneys of the BAFF-R strain.

Il1rn showed a slightly lower expression level in BAFF knockout kidneys compared to wildtype kidneys (Fig 5B). The Il1rn expression was higher in BAFF-R knockout kidneys compared to wildtype kidneys. Ischemic wildtype kidneys of the BAFF and BAFF-R strain showed a similar expression of Il1rn. For all contralateral kidneys no Il1rn expression was detected.

While Lcn2, Lyz2, Cd44, Fn1 and Il1rn were upregulated in BAFF knockout kidneys after ischemia/reperfusion, the expression level was consistently lower compared to wildtype littermates. Ischemic BAFF-R knockout kidneys showed upregulated expression of Lcn2, Lyz2, Cd44, Fn1 and Il1rn as well. However, expression levels were always higher in knockout compared to wildtype kidneys. Regarding Havcr1 expression pattern we found the exact opposite: higher Havcr1 expression in BAFF knockout than wildtype kidneys and lower expression of Havcr1 in BAFF-R knockout kidneys compared to wildtype littermates.

One of the downregulated genes due to ischemia/reperfusion injury was Kl (Fig 5C). The expression of Kl was higher in ischemic BAFF knockout kidneys compared to those of wildtype littermates, whereas BAFF-R knockout kidneys showed lower Kl expression than the wildtype. Expression level of Kl in contralateral kidneys was nearly the same, except BAFF-R wildtype kidneys showing slightly higher expression.

Expression of Kl was downregulated in BAFF as well as BAFF-R knockout kidneys after ischemia/reperfusion. But expression level of knockout kidneys was higher compared to wildtype littermates in case of the BAFF strain and lower in BAFF-R strain.

**Fig 6:**
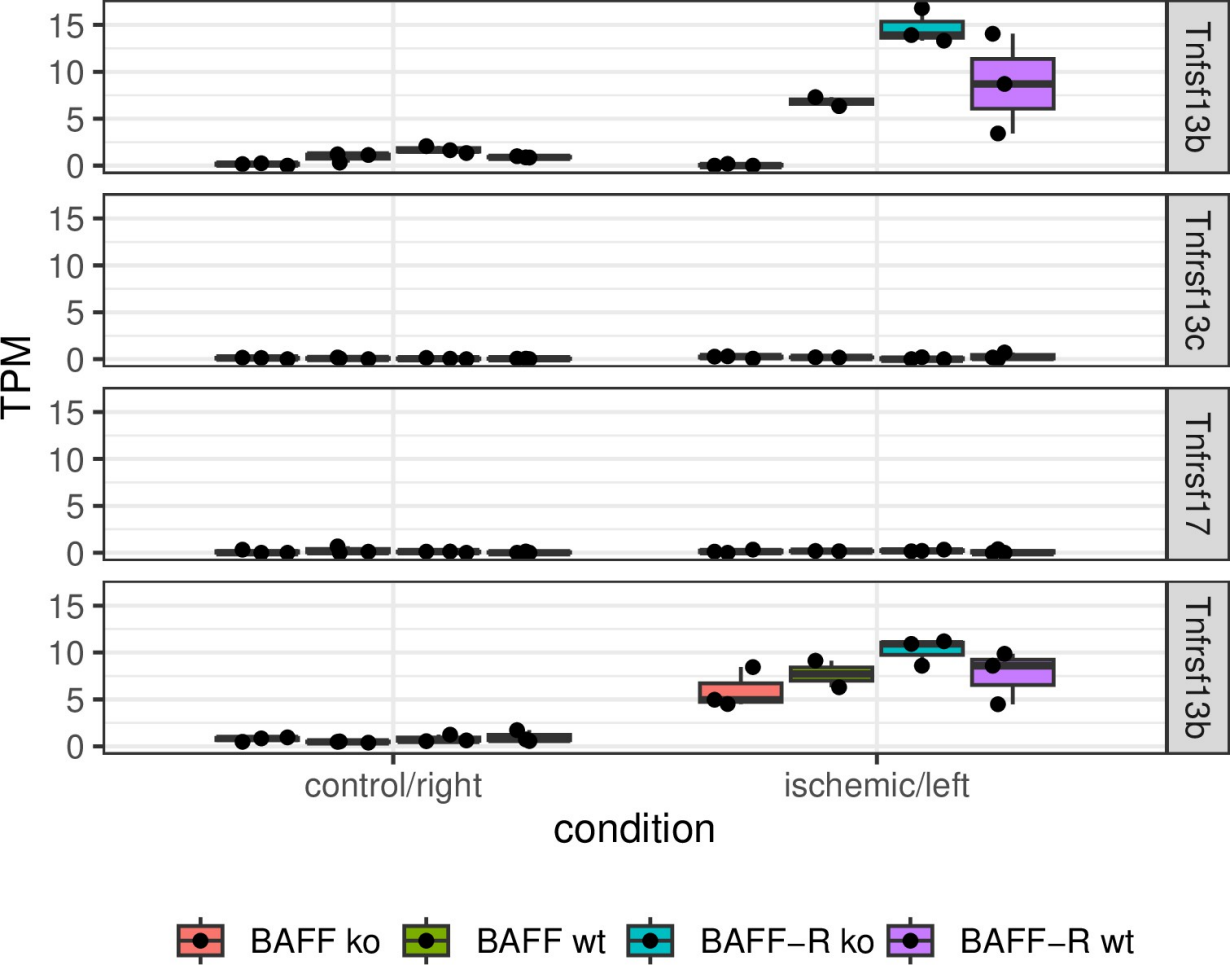
Transcripts per million (TPM) of analyzed genes of ischemic kidneys compared to contralateral control in BAFF (B6.129S2-*Tnfsf13b*^*tm1Msc*^/J) and BAFF-R (B6(Cg)-*Tnfrsf13c*^*tm1Mass*^/J) strain, in each case distinguished between knockout (ko) and wildtype (wt) mice. Tnfsf13b: BAFF, Tnfrsf13c: BAFF-R, Tnfrsf17: BCMA, Tnfrsf13b: TACI. Each point shows one sample.

BAFF (Tnfsf13b) expression itself was upregulated in ischemic kidneys (Fig 6). Expression level was higher in BAFF-R knockout kidneys in comparison with the wildtype. With regard to the different receptors of BAFF, no BAFF-R (Tnfrsf13c) expression has been observed at all for the majority of mice and only very low transcript counts (2 TPM) in one mouse (Fig 6). Also, no BCMA (Tnfrsf17) expression was detected, neither in kidneys of BAFF nor BAFF-R strain, again with the exception of very low number of detected transcripts in one mouse (3 TPM) (Fig 6). Only TACI (Tnfrsf13b) was expressed in ischemic kidneys (Fig 6). TACI expression was slightly lower in BAFF knockout compared to wildtype ischemic kidneys. BAFF-R knockout ischemic kidneys showed slightly higher expression level of TACI than wildtype ischemic kidneys.

## Discussion

We performed renal unilateral ischemia/reperfusion in BAFF and BAFF-R knockout mice and analyzed the transcriptome of ischemic as well as contralateral kidneys after a reperfusion time of three days in order to get a comprehensive overview of regulated genes involved in the pathophysiology of acute kidney injury. As expected, I/R injury led to an upregulation of the cytokine BAFF, supporting the hypothesis that BAFF is involved in the pathophysiology of AKI. This is in line with several other findings in view of BAFF expression and AKI as well as kidney transplantation [17–22]. Interestingly, BAFF expression seems to be higher in ischemic kidneys of BAFF-R knockout mice compared to wildtype littermates. It is conceivable that missing signaling via BAFF-R enhances BAFF expression. However, not only in BAFF-R knockout kidneys, also in BAFF knockout and wildtype kidneys of both strains there was no BAFF-R expression. This contradicts the assumption of a higher BAFF expression due to the BAFF-R knockout. This finding is consequently only due to the strain differences. BCMA was also not expressed in kidneys of both strains, but TACI. This BAFF receptor was expressed in BAFF knockout as well as wildtype ischemic kidneys at nearly the same level. BAFF-R knockout ischemic kidneys showed slightly higher TACI expression compared to wildtype ischemic kidneys. TACI, overexpressed in patients with chronic antibody-mediated rejection, is discussed as potential biomarker for distinguishing these patients from stable ones [17]. Our finding shows a potential involvement of TACI during the acute phase after I/R injury and therefore eventually a role of TACI in AKI.

The transcriptome analyses revealed four genes, Havcr1, Lcn2, Lyz2 and Cd44, upregulated in both strains regardless of the genotype. All of them rank among the top 40 differentially expressed genes.

Hepatitis A virus cellular receptor 1 (abb. and gene name: Havcr1), also known as Kidney Injury Molecule-1 (KIM-1), is a proximal tubule apical transmembrane protein. Under physiological conditions levels of Havcr1 are low. Increased expression was detected in TEC of biopsies and urine from patients with acute tubular necrosis [61], 48 hours after unilateral I/R in proximal TECs of rats [62] and correlated with inflammation, fibrosis and renal function [63]. Different studies showed that Havcr1 is closely linked with AKI [61, 64–69].

As might be expected, Havcr1 was under the top 40 differentially expressed genes of ischemic kidneys in both mouse strains in our study. In order to compare both strains, we detected a different expression pattern. Expression of Havcr1 was higher in BAFF knockout than wildtype kidney, but lower in BAFF-R knockout kidneys compared to wildtypes. The different Havcr1 expression patterns of BAFF and BAFF-R strain are a first indication of a different impact of BAFF and BAFF-R knockout with regard to I/R injury.

Neutrophil Gelatinase-Associated Lipocalin (NGAL, gene name: Lcn2) is expressed in different tissues and one of the strongest induced genes after renal I/R [70]. NGAL was identified as a potential biomarker for the initiation phase of acute kidney injury in a genome-wide interrogation strategy to identify renal genes with early induction in animal models [31, 71]. It is upregulated in proximal TECs and distal nephron segments [70] and it is assumed, that damaged TECs express NGAL in order to induce re-epithelialization [72].

In the present data an upregulation of NGAL in ischemic kidneys of both strains is shown. Furthermore, we detected a different expression pattern in the BAFF and BAFF-R strain. The expression level of Lcn2 was lower in BAFF knockout ischemic kidneys compared to the wildtype, but in case of BAFF-R knockout the Lcn2 expression was higher compared to ischemic wildtype kidneys. This could be a further indication of a different impact of BAFF and BAFF-R knockout in this I/R model, even if Lcn2 expression pattern of both strain is the exact opposite of the detected Havcr1 expression pattern.

Lysozyme (gene name: Lyz, in this study examined: Lyz2) is in its function as a bacteriolytic enzyme an important component of the innate immune system. In 1964 the study of Prockop and Davidson [73] was the first attempt to correlate urinary lysozyme excretion with its serum levels and parameters of renal function. The study also examined the effect of glomerular or tubular damage on lysozyme excretion and led to the assumption, that lysozymuria may be a useful indicator of renal tubular damage and serves as a diagnostic as well as prognostic tool [73]. Alterations in urine and serum levels of lysozyme were detected in acute renal failure [74]. Up to 1988 several publications discussed the occurrence and role of lysozyme in context of renal (transplant) damage and failure, also in view of suitability as diagnostic marker [75–83]. First report, that lysozyme, intravenously infused into male rats, induced functional as well as structural alterations and finally caused acute renal failure was presented by Cojocel *et al*. [84]. In 2009 a case report by Patel *et al*. [85] reminded of the nephrotoxic effect of lysozyme and kidney failure as common feature in hematological malignancies, especially chronic myelomonocytic leukemia (CMML). Overproduction of lysozyme by tumoral cells in CMML patients causes acute kidney injury based on lysozyme-induced proximal tubular damage [86–88]. In spite of all these findings in almost 60 years and a huge gap since the 90’s, lysozyme seems to have been forgotten as potential early biomarker in acute kidney injury. In case of chronic kidney disease (CKD), lysozyme was measured among other CKD-associated plasma proteins in a multiple reaction monitoring (MRM) mass spectrometry (MS) assay in order to determine their association with kidney function and disease outcome [89].

To our knowledge, this study is the first report of upregulated Lyz2 expression in a murine ischemia/reperfusion model of the kidneys.

In addition, not only the expression of Lysozyme 2 was detected in knockout as well as wildtype ischemic kidneys of both strains, but also a difference in expression level due to BAFF and BAFF-R knockout was investigated. Lysozyme 2 expression was lower in case of BAFF knockout, while BAFF-R knockout kidneys showed higher expression levels compared to their respective wildtype littermates. This finding is comparable to the results for Lcn2 expression.

CD44 is a glycoprotein important for cellular migration, adhesion and interepithelial cell interactions [90–92]. Upregulation of CD44 expression in glomeruli and TEC is shown in case of inflammatory and autoimmune driven renal diseases [93–96]. Furthermore, expression levels are increased in the early phase after I/R injury [97] and renal damage is caused by the contribution of CD44 to the migration of neutrophils into postischemic kidneys [98].

In our study, the expression of Cd44 was lower in BAFF knockout than wildtype kidneys, but higher in BAFF-R knockout kidneys compared to wildtypes. This is in line with the results of Lcn2 and Lyz2 expression.

We also investigated the expression pattern of the further upregulated gene Fn1 in ischemic compared to contralateral kidneys. Fibronectin 1 (gene name: Fn1) is a glycoprotein which is involved in cellular adhesion, growth and angiogenesis [reviewed in 99]. For the I/R model of the heart is shown, that Fibronectin 1 is upregulated and its inhibition leads to a reduction of myocardial infarct size. Myocyte apoptosis, inflammation, oxidative stress and fibrosis were ameliorated [100].

The present study has shown, that Fibronectin 1 is upregulated in ischemic kidneys of knockout and wildtype mice of B6.129S2-*Tnfsf13b*^*tm1Msc*^/J and B6(Cg)-*Tnfrsf13c*^*tm1Mass*^/J strains. As already shown for Lcn2, Lyz2 and Cd44, there is also a different expression pattern for Fn1 in BAFF and BAFF-R knockout ischemic kidneys compared to the corresponding wildtypes. Fn1 expression was lower in BAFF knockout than wildtype, but higher in BAFF-R knockout compared to wildtype mice.

Binding of the Interleukin-1 receptor antagonist (IL-1RA, gene name: Il1rn) to the Interleukin-1 receptor (IL-1R) inhibits signaling of Interleukin-1 (IL-1), which contributes to inflammation and apoptosis [101, 102]. It is shown that the administration of recombinant IL-1ra attenuates renal I/R injury [103].

With regard to ischemic kidneys, BAFF knockout showed slightly lower Il1rn expression levels compared to the wildtype, whereas expression of Il1rn was higher in BAFF-R knockout compared to wildtypes. Results are comparable with expression pattern of Lcn2, Lyz2, Cd44 and Fn1.

Finally, we investigated the expression of one of the downregulated genes called Kl in both strains. Kidneys exhibit the highest expression levels of *α*-Klotho and seems to be the main source of soluble Klotho in circulation [104]. *α*-Klotho regulates the calcium and phosphate transport in kidneys and acts as a co-receptor for Fibroblast Growth Factor 23 (FGF23) [105, 106]. In general, levels of *α*-Klotho are low during AKI [107, 108]. Kidney damage is caused by renal apoptosis and calcification in order to a loss of *α*-Klotho [109]. In ischemia/reperfusion injury *α*-Klotho levels decrease [108, 110].

In our study we confirmed the finding of *α*-Klotho reduction after I/R injury in ischemic kidneys of both strains compared to contralateral controls. Interestingly, the observed expression pattern in case of Lcn2, Lyz2, Cd44, Fn1 and Il1rn was also shown for Kl in BAFF and BAFF-R knockout ischemic kidneys in opposite manner. Expression of Kl was higher in BAFF knockout ischemic compared to wildtype kidneys, but lower in BAFF-R knockout compared to wildtype kidneys. Therefore, in case of all six differentially expressed genes Lcn2, Lyz2, Cd44, Fn1, Il1rn and Kl the same expression pattern for BAFF and BAFF-R knockout was observed, which supports the cautious assumption that the knockout of BAFF and BAFF-R has an impact on I/R injury and thereby maybe a different effect.

Overall, the study reveals a uniform pattern for BAFF and BAFF-R knockout with regard to the expression levels of the genes Lcn2, Lyz2, Cd44, Fn1, Il1rn and Kl, which were studied in more detail. In case of upregulated genes Lcn2, Lyz2, Cd44, Fn1 and Il1rn their expression levels were higher in BAFF-R knockout and lower in BAFF knockout ischemic kidneys compared to wildtype littermates. The expression level of the downregulated gene Kl was lower in BAFF-R knockout and higher in BAFF knockout kidneys after I/R compared to wildtype littermates. This leads to the assumption that damage induced by ischemia/reperfusion is reduced in BAFF knockout mice, but enlarged in BAFF-R knockout. Thus, it could be speculated that the cytokine BAFF may have a negative effect on kidneys undergoing I/R. Based on the findings for the BAFF-R knockout there must be another signaling pathway for BAFF distinct from BAFF-R. BAFF may signal via BCMA or TACI. In our analyses no expression of BCMA could be detected. This indicates that signaling of BAFF via TACI may play a role in this I/R model. In addition, renal injury seems to be increased in BAFF-R knockout compared to wildtype littermates. If BAFF does not signal via BAFF-R, extent of damage must be the same in BAFF-R knockout and wildtype mice. A possible explanation is the signaling of BAFF via different receptors with contrary effects. BAFF could possibly signal via BAFF-R and thereby mediates positive effects. This would explain why wildtype ischemic kidneys show less damage than BAFF-R knockout ischemic kidneys. It is conceivable that BAFF has an ambivalent role in AKI induced by I/R injury.

At first sight it seems to be contradictory to assume a positive effect of BAFF knockout and a negative one for BAFF-R knockout with regard to I/R injury even though Havcr1 expression is higher in BAFF knockout and lower BAFF-R knockout compared to their respective wildtype littermates. However, it is not possible to deduce the effect of both knockouts from Havcr1 expression levels. We can detect the expression level in our study, but exact impact of Havcr1 upregulation in this I/R model remains unclear due to the versatile function of KIM-1 in renal tubular damage. It is known that KIM-1 mediates the phagocytosis of apoptotic cells during AKI and therefore protects kidneys by downregulation of NF-*κ*B, which leads to a decreased inflammation [111]. Otherwise, KIM-1 activates inflammatory signaling in TEC without an injury stimulus by itself and therefore provokes renal inflammation [112]. Chen *et al*. [113] showed in their study that hypoxia-treated TECs released small extracellular vesicles (sEVs), which were taken up by KIM-1 expressing TEC. KIM-1 can recognize Phosphatidylserine on the surface of sEV and therefore act as a membrane receptor. In this manner tubulointerstitial inflammation induced by hypoxia is amplified [113]. It is not clear which signals decide whether KIM-1 has a protective effect on injured kidneys or not. Without knowing the exact signaling of KIM-1 in our study, the higher expression of Havcr1 in BAFF knockout compared to wildtype ischemic kidneys cannot be finally assessed. If we assume that KIM-1 had a protective function in our I/R model, BAFF knockout would enforce this positive impact compared to wildtype littermates and BAFF-R knockout would reduce it. This would be in line with our remaining findings.

Nevertheless, based on this initial study, further investigations with larger sample sizes are needed to rule out that present findings are not only explained by strain differences and that detection is not restricted to the RNA level and therefore be also detectable on protein level. In addition, only female mice were analyzed in this study. Therefore, it must be considered that female hormone cycle effected the revealed results. As an initial assessment we analyzed the expression of the key molecules along the hypothalamic-pituitary-gonadal axis in our samples and observed no expression or only low transcript counts (S2 Fig). We therefore argue, that the identified RNA abundance, if not only background, is too low to play any meaningful role. Thus, female hormone levels do not seem to be the predominant factors in biasing the results of this study. Furthermore, it is not clear why the expression pattern of the approved biomarker Havcr1 is the exact opposite of the identical expression pattern of Lcn2, Lyz2, Cd44, Fn1, Il1rn and Kl in both strains and consequently what does it mean for the effect of BAFF and BAFF-R knockout in I/R injury. We have no clear causal link between upregulated Havcr1 expression and BAFF knockout.

## Conclusion

We can summarize that in case of all well-established as well as potential early I/R biomarker an up- or downregulation was detected in ischemic kidneys of both strains. Furthermore, upregulation of Lcn2, Lyz2, Cd44, Fn1 and Il1rn as well as downregulation of Kl were higher in case of BAFF-R knockout and lower in BAFF knockout compared to corresponding wildtype littermates in a consistent manner. This leads to the assumption that BAFF knockout has a positive effect on kidneys undergoing I/R, while BAFF-R knockout worsened the renal damage after I/R. Against the background that BAFF KO mice analyzed in this study are immunodeficient with a significant loss of mature B cells and attenuated T cell-dependent as well as T cell-independent antibody response, assumed positive effect of BAFF knockout in case of I/R injury seems to have a strong connection with immunological signaling. Therefore, it would be interesting to compare the results of this unilateral, not systemic renal damage model with renal injury provoked by an inflammatory and systemic stimulus in future investigations. It is additionally worth to point out the remarkable Lysozyme 2 expression in ischemic kidneys of both strains. Further studies should be addressed to investigate a potential use of Lysozyme as predictive and early biomarker for AKI.

## Supporting information

S1 Fig Principal component analysis (PCA) of ischemic and contralateral control kidneys. BAFF (B6.129S2-*Tnfsf13b*^*tm1Msc*^/J) and BAFF-R (B6(Cg)-*Tnfrsf13c*^*tm1Mass*^/J) strain.
(TIF)

S2 Fig Transcripts per million (TPM) of genes along hypothalamic-pituitary-gonadal axis. Analyzed in ischemic kidneys compared to contralateral control in BAFF (B6.129S2-*Tnfsf13b*^*tm1Msc*^/J) and BAFF-R (B6(Cg)-*Tnfrsf13c*^*tm1Mass*^/J) strain, in each case distinguished between knockout (ko) and wildtype (wt) mice. Esr1: estrogen receptor 1, Pgr: progesterone receptor, Lhb: luteinizing hormone subunit beta, Fshb: follicle stimulating hormone subunit beta, Cga: glycoprotein hormones alpha chain, Fshr: follicle stimulating hormone receptor.
(TIF)

S1 File This file contains all differentially expressed genes with adjusted p-value < 0.05 and log2foldchange > 1, < -1 resp. File named ‘DE_results_filtered’ and is additionally available at https://github.com/sebboegel/moeckel2022.
(CSV)
