# Supplementary figures and images for: Transcriptome analysis of renal ischemia/reperfusion (I/R) injury in BAFF and BAFF-R deficient mice
Source: PLoS One. 2023 Sep 26;18(9):e0291619. doi: 10.1371/journal.pone.0291619 (PMC10522044; doi:10.1371/journal.pone.0291619)

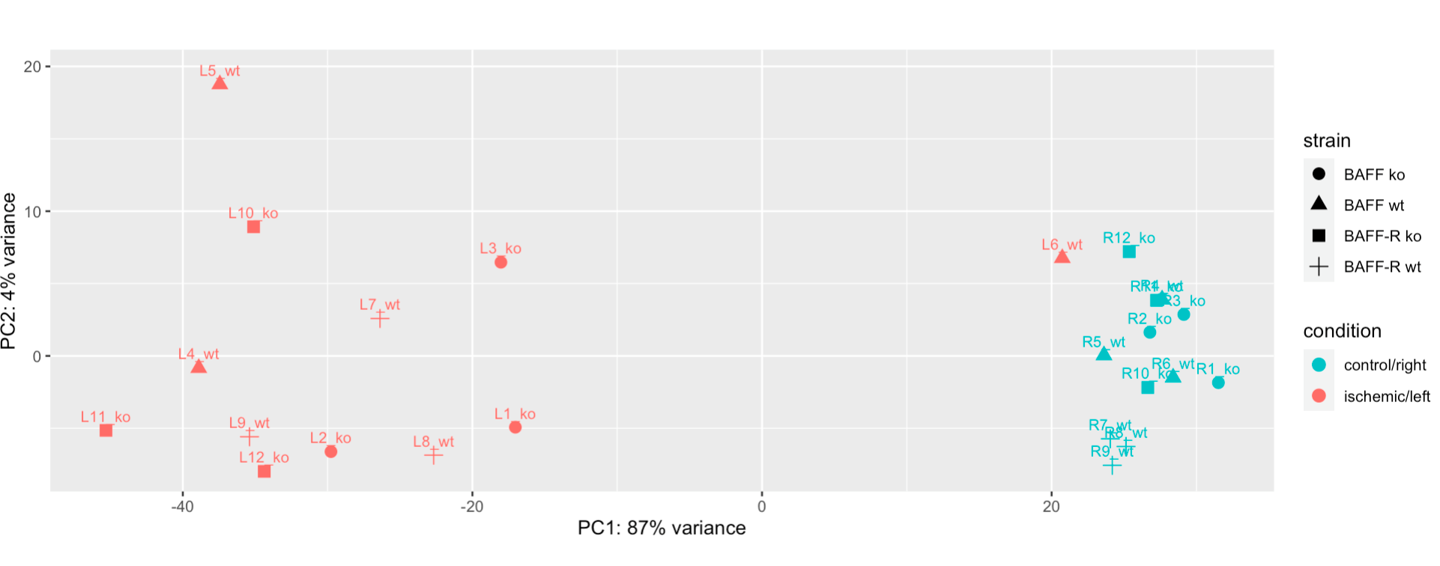

Supplement: S1 Fig — BAFF (B6.129S2-Tnfsf13btm1Msc/J) and BAFF-R (B6(Cg)-Tnfrsf13ctm1Mass/J) strain. (TIF) [file pone.0291619.s001.tif]

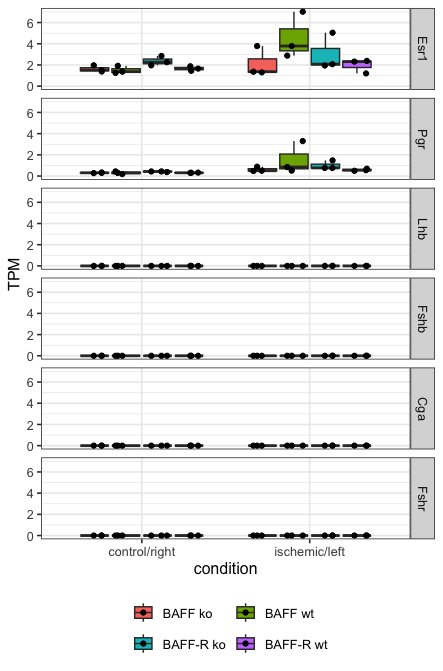

Supplement: S2 Fig — Analyzed in ischemic kidneys compared to contralateral control in BAFF (B6.129S2-Tnfsf13btm1Msc/J) and BAFF-R (B6(Cg)-Tnfrsf13ctm1Mass/J) strain, in each case distinguished between knockout (ko) and wildtype (wt) mice. Esr1: estrogen receptor 1, Pgr: progesterone receptor, Lhb: luteinizing hormone subunit beta, Fshb: follicle stimulating hormone subunit beta, Cga: glycoprotein hormones alpha chain, Fshr: follicle stimulating hormone receptor. (TIF) [file pone.0291619.s002.tif]
